# Bayesian StairwayPlot for Inferring Single Population Demographic Histories From Site Frequency Spectra

**DOI:** 10.1111/1755-0998.14087

**Published:** 2025-02-26

**Authors:** Sebastian Höhna, Ana Catalán

**Affiliations:** ^1^ GeoBio‐Center LMU Ludwig‐Maximilians‐Universität München Munich Germany; ^2^ Department of Earth and Environmental Sciences, Paleontology and Geobiology Ludwig‐Maximilians‐Universität München Munich Germany; ^3^ Division of Evolutionary Biology Ludwig‐Maximilians‐Universität München Munich Germany

**Keywords:** coalescent, RevBayes, whole genome SNP data

## Abstract

The StairwayPlot approach provides an elegant, flexible and powerful method to estimate complex demographic histories of single populations from site frequency spectrum data. It uses expected coalescent times to compute the expected site frequency spectrum within a multinomial likelihood function. Population sizes are allowed to vary freely between coalescent events but are constant within each interval. Here, we implement the StairwayPlot approach in the Bayesian software package RevBayes. We use approaches developed for Bayesian Skyline Plots, which include independent and identically distributed (i.i.d.) population sizes, Gaussian Markov random fields and Horseshoe Markov random fields as prior distributions on population sizes. Furthermore, we implement a recently developed approach for computing the leave‐one‐out cross‐validation probability for efficient model selection. We compare inference from our Bayesian implementation to the original Maximum Likelihood implementation, StairwayPlot2. Our results show that our Bayesian implementation in RevBayes performs comparable to StairwayPlot2 in terms of parameter accuracy, which is expected given that both use the same underlying likelihood function. From our set of prior models, the Gaussian Markov random field prior performed best for smoothly varying demographic histories, while the Horseshoe Markov random field performs best for abruptly changing demographic histories. We conclude the study by exploring several choices often faced in empirical studies, including the estimate of the total sequence length, the assumed mutation rate, as well as biases through mis‐calling ancestral alleles. We show using our empirical example that as few as 10 diploid individuals are sufficient to infer complex demographic histories, but at least 500 k single nucleotide polymorphisms (SNPs) are required.

## 1 Introduction

The demographic history of a species represents one of the most fundamental and baseline parameters of population genetics (Li and Stephan 2006; Marchi et al. 2021). In some cases, the demographic history of a species is the focus of the study, for example, if the population underwent a recent expansion, decline or a bottleneck. In other cases, the demographic history represents the null hypothesis to study adaptation (Johri et al. 2022).

In the last decades, several different approaches for inferring demographic histories have been developed. These approaches can broadly be classified into (i) coalescent‐based inferences using genealogies of one or multiple loci (Pybus et al. 2000), (ii) sequential Markovian coalescent‐based methods using genomes of a single individual (PSMC, Li and Durbin 2011) or multiple individuals (MSMC, Schiffels and Durbin 2014), (iii) ancestral recombination graph‐based methods using genomes of multiple individuals (Rasmussen et al. 2014; Kelleher et al. 2019), (iv) SNP‐based methods using the site or allele frequency spectrum (Gutenkunst et al. 2009; Liu and Fu 2015), and (v) summary statistic‐based methods using approximate Bayesian computation (ABC, Beaumont et al. 2002; Marjoram et al. 2003). Each approach has different underlying model assumption, e.g., independence among SNPs or complete linkage of sites within a locus, while other approaches try to model recombination. Furthermore, each approach has different requirements and assumptions of the data, e.g., full (phased) genomes, only SNPs or single/multiple loci. We refer the reader to several reviews on the topic for more details (Beaumont 2010; Ho and Shapiro 2011; Beichman et al. 2018; Mather et al. 2020; Nielsen et al. 2024). Here, we focus on SNP‐based demographic inference from whole genome data, specifically adapting the StairwayPlot approach (Liu and Fu 2015).

The StairwayPlot approach is an efficient and flexible demographic inference method that uses the site frequency spectrum and thus can be readily applied to many population genomics datasets (Liu and Fu 2015, 2020; Liu 2020). Specifically, the StairwayPlot approach is useful for datasets with many individuals and whole genome resequencing approaches (Lou et al. 2021). The probability of the observed site frequency spectrum is given by a multinomial distribution (i.e., composite likelihood) parameterized with the vector of site frequency probabilities. The site frequency probabilities are computed given the expected coalescent times and constant population sizes between coalescent events. Thus, the StairwayPlot approach can estimate independent population sizes between each expected coalescent event, or group these estimates for improved parameter estimation. Furthermore, Liu and Fu (2015) suggest that ‘one possible improvement for the stairway plot with respect to the estimation of ancient histories is achieved by integrating the composite likelihood into a Bayesian framework, which smoothens the θ estimations into continuous probability estimations.’ We will present here such a Bayesian implementation of the StairwayPlot approach and evaluate our implementation against the original StairwayPlot2 (Liu and Fu 2020).

Inferring complex demographic histories using Bayesian approaches has been successfully and extensively applied using Bayesian skyline plots over the last two decades (Ho and Shapiro 2011). Bayesian skyline plots use coalescent likelihoods and either genealogies as data directly or multiple sequence alignments to infer the genealogy and demographic history jointly (Drummond et al. 2005; Billenstein and Höhna 2024). Much of the recent work on Bayesian skyline plots has focused on building better prior models to model population size changes through time (Gill et al. 2013; Faulkner et al. 2020; Billenstein and Höhna 2024). The first skyline plot method, which was not Bayesian, inferred one population size per coalescent interval (Pybus et al. 2000). However, this method had the tendency to overestimate variation in population sizes and therefore was extended to group neighbouring coalescent intervals to receive the same population size parameter (Strimmer and Pybus 2001). The first Bayesian skyline plot method also assigned neighbouring coalescent intervals the same population size but assumed the number of groups to be defined a priori, while the size of each group was estimated (Drummond et al. 2005). The pre‐defined number of groups was relaxed in the extended Bayesian skyline plot where neighbouring intervals were merged or split during the analysis, thus allowing for a flexible number of groups (Heled and Drummond 2008). Other recent extensions kept the number of population size parameters equal to the number of coalescent events and applied smoothing priors (Minin et al. 2008; Gill et al. 2013; Faulkner et al. 2020). Specifically, the Gaussian Markov random field (GMRF) priors assume that population sizes change according to a normal distribution, thus assuming that population sizes change over time according to a Brownian motion or geometric Brownian motion (Minin et al. 2008; Gill et al. 2013). Similarly, the Horseshoe Markov random field (HSMRF) assumes that consecutive population sizes change according to a normal distribution where the variance can change, thus extending the GMRF to allow for jumps (Faulkner et al. 2020; Magee et al. 2020).

Here, we implement the underlying likelihood function of the StairwayPlot approach (Liu and Fu 2015, 2020) into the Bayesian software package RevBayes (Höhna et al. 2016). RevBayes provides all commonly used prior distributions for Bayesian skyline plots (Billenstein and Höhna 2024), which can readily be applied in combination with our newly implemented StairwayPlot likelihood function. Furthermore, we implement a recently developed approach for model selection via leave‐one‐out cross‐validation that can be computed efficiently from the samples of the posterior distribution (Lartillot 2023). We explore and evaluate our new Bayesian implementation on standard simulated datasets and discuss specific extensions, such as folding the site frequency spectrum, the number of monomorphic sites (or total sequence length) and uncertainty in mutation rates using an empirical example of the big European firefly ( *Lampyris noctiluca* ) (Catalan et al. 2024). Finally, we explore several choices faced in empirical analyses, including (i) the impact of the assumed total sequence length, (ii) the assumed mutation rate, (iii) the number of sequenced individuals, (iv) the number of SNPs obtained and (v) the impact of incorrectly assigning derived and acenstral alleles.

## 2 Material and Methods

### 2.1 The StairwayPlot Approach

The StairwayPlot approach (Liu and Fu 2015, 2020) follows common population genetic models assuming a coalescent process and an infinite sites model. The important aspect of the StairwayPlot approach is the flexibility of parameter specification (the demographic history) and how the likelihood function is computed. Here we briefly present the StairwayPlot approach, the model assumptions and likelihood function derivation, following Liu and Fu (2015) and Liu and Fu (2020).

#### 2.1.1 Likelihood Function

We start with some important modelling assumptions. Let us assume that we have a sample from m diploid individuals from a panmictic population, thus representing n=2m haploid individuals. Furthermore, let us assume that the relationship among the n individuals can be modelled as a standard coalescent process (Kingman 1982) and that mutations are rare, i.e., at most one mutation can occur at a given site. For mathematical convenience, we assume that population sizes change at (expected) coalescent events (following Liu and Fu 2015), that is, population sizes are constant between coalescent events but can change arbitrarily between coalescent intervals. This assumption is clearly unrealistic and actually mathematically inconsistent (Billenstein and Höhna 2024); however, the approximation works reasonably well in practice (Pybus et al. 2000; Drummond et al. 2005; Heled and Drummond 2008). For simplicity, we also assume that each site is independent, unlinked and neutrally evolving.

Next, the observed data are summarised by the site frequency spectrum, which is the count of how often we observed a site with i derived alleles and n−i ancestral alleles. The likelihood is then computed as the probability of observing the given site frequency spectrum using a composite likelihood function (which here is equivalent to the multinomial likelihood). In spirit, the probability of a specific site frequency is given by the probability of the coalescent tree and that a mutation has occurred on a lineage with exactly i descendants. Therefore, we need to specify two probabilities: (i) the probability of the length of the lineage and (ii) the probability of this lineage having i descendants. Both probabilities are given by the coalescent process. Moreover, we assume that at most one mutation can occur and the probability of the mutation occurring at this branch is proportional to the length of the branch.

Let us recall the probability density of the *k*th coalescent time $t_{k}$ (Kingman 1982) where k is the current number of samples that can coalesce in a population of effective size $N_{k}$,

(1) $$P\left(t_{k}|N_{k}\right)=\frac{\left(\begin{array}{l} k\\ 2 \end{array}\right)}{2N_{k}}\exp\left(-\frac{\left(\begin{array}{l} k\\ 2 \end{array}\right)}{2N_{k}}t_{k}\right)$$

Hence, this coalescent time $t_{k}$ is exponentially distributed with rate $\frac{k\left(k-1\right)}{4N_{k}}$. Instead of using the actual probability densities and estimating each coalescent time separately, or integrating over it, we use directly the expected coalescent time

(2) $$E\left[t_{k}|N_{k}\right]=\frac{4N_{k}}{k\left(k-1\right)}$$

Next, the probability $\mathrm{Pr}\left(k,i|n\right)$ that a lineage has exactly i sampled descendants when there are currently k lineages with sampled descendants and a total of n samples at the present is given by the combinatorial expression (Fu 1995)

(3) $$\mathrm{Pr}\left(k,i|n\right)=\left\{\begin{array}{ll} \frac{\left(\begin{array}{c} n-i-1\\ k-2 \end{array}\right)}{\left(\begin{array}{c} n-1\\ k-1 \end{array}\right)} & \text{if}\;n-i+1\geq k\geq 2\;\text{and}\;n\geq i\geq 1\\ 0 & \text{otherwise} \end{array}\right.$$

The probability $p_{i}$ of i individuals to have the allele with the mutation is composed of: (i) k lineages with sampled descendants were present, (ii) any of the k lineages could have experienced the mutation, (iii) the length of time that there are k lineages and (iv) any valid choice of k≥i and k≤n. Thus, the probability $p_{i}$ is computed by (Liu and Fu 2015, 2020)
(4)

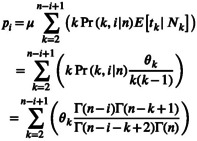

where $\theta_{k}=4N_{k}\mu$. It is possible that we obtain nonsensically large values for $p_{i}$ if $\theta_{i}$ is unrealistically large. For example, we would expect more than one mutation for large values of $\theta_{i}$, which would violate the infinite sites model assumption. Therefore, we need to enforce that $\sum_{i=1}^{n-1}p_{i}<1$ and simply set the log‐likelihood to −Inf otherwise.

Note that we cannot use the same approach to compute to the probability $p_{0}$ for monomorphic sites. The original implementation (Liu and Fu 2015) suggested to use

(5) $$p_{0}=1-\sum_{i=1}^{n-1}p_{i}$$

Alternatively, we could compute the probability of no mutation occurring over the entire coalescent tree length T, which has the expected value
(6)

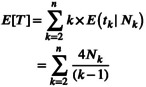

and thus gives the probability of observing no mutation by

(7) $$p_{0}=e^{-\sum_{k=2}^{n}\frac{\theta_{k}}{\left(k-1\right)}}$$

In this case, we need to normalise the allele frequency probability $p_{i}$ so that $\sum_{i=1}^{n-1}p_{i}=1-p_{0}$. Similarly, we cannot compute the probability $p_{n}$ of all individuals having the derived allele because this would require at least two mutations, assuming that the common ancestor of all individuals did not have the derived allele. We explore the impact of choosing either the previously suggested approach (Equation 5) and our new approach (Equation 7) for computing the probability of monomorphic sites in Figure S4.

Finally, we compute the likelihood function of θ, the vector of coalescent parameters, as the composite likelihood function, i.e., multinomial likelihood

(8) $$L\left(\theta \right)=l!\prod_{i=0}^{n-1}\frac{p_{i}^{\xi_{i}}}{\xi_{i}!}$$

where l is the total number of sites and $\xi_{i}$ is the frequency of observing allele count i, with $l=\sum_{i=1}^{n-1}\xi_{i}$. Note that Equations ((1), (2), (3), (4), (5)–(1), (2), (3), (4), (5)) and (8) are equivalent to those reported in Liu and Fu (2015) and Liu and Fu (2020).

#### 2.1.2 Ascertainment Bias Correction for Not Observing Monomorphic or Singleton Sites

In some situations, it might be necessary to adjust the likelihood because not all allele frequencies could be sampled. For example, many recent genomic SNP datasets, such as those obtained from ddRadSeq, do not contain reliable information about monomorphic sites (e.g., Catalan et al. 2022). Furthermore, singleton sites, i.e., sites with an allele frequency of i=1, are more prone to sequencing errors (Duchen et al. 2013). It might therefore be advisable to exclude these allele frequencies from the study.

We modify the likelihood to condition on not observing monomorphic sites using an ascertainment bias correction (Felsenstein 1992; Lewis 2001). Specifically, we modify the probability $p_{i}$ of observing a given allele count to $p_{i}^{M}=\frac{p_{i}}{1-p_{0}}$ and then obtain the ascertainment bias corrected likelihood by

(9) $$L^{M}\left(\theta \right)=l_{\left(-0\right)}!\prod_{i=1}^{n-1}\frac{{\left(p_{i}^{M}\right)}^{\xi_{i}}}{\xi_{i}!}$$

where $l_{\left(-0\right)}$ is the total number of sites without monomorphic sites.

Similarly, we modify the likelihood function to condition on observing neither monomorphic nor sites that carry only one copy of either the derived or ancestral allele by replacing the probability $p_{i}$ of observing a given allele count with $p_{i}^{S}=\frac{p_{i}}{1-p_{0}-p_{1}-p_{n-1}}$, which yields

(10) $$L^{S}\left(\theta \right)=l_{\left(-0,1,n-1\right)}!\prod_{i=2}^{n-2}\frac{{\left(p_{i}^{S}\right)}^{\xi_{i}}}{\xi_{i}!}$$

where $l_{\left(-0,1,n-1\right)}$ is the total number of sites without monomorphic and sites that carry only one copy of either the derived or ancestral allele.

#### 2.1.3 Likelihood Function for the Folded Site Frequency Spectrum

In many cases, we do not have information about derived vs. ancestral alleles, for example, we do not have information about the outgroup population. In these situations, we need to use the folded site frequency spectrum. Our previous likelihood functions can be modified to apply to the folded site frequency spectrum by

(11) $$L^{F}\left(\theta \right)=l!\prod_{i=0}^{n/2}\frac{{\left(p_{i}^{F}\right)}^{\xi_{i}}}{\xi_{i}!}$$

where
(12)

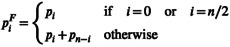




Ascertainment bias corrections for not observing monomorphic or singleton sites can be applied to the likelihood function of the folded site frequency spectrum.

### 2.2 Prior Models

The strength of a Bayesian analysis lies in its ability to specify prior knowledge via prior distributions. In the context of Bayesian skyline plots, this prior knowledge is specified using shrinkage or smoothing priors (Gill et al. 2013; Magee et al. 2020). A shrinkage or smoothing prior has the goal to regularise a model with many parameters to obtain smooth estimates. A model with many parameters, such as one population size parameter per coalescent interval, might be over‐parameterised and the shrinkage prior pulls the model to collapse or shrink towards a simpler model with fewer parameters (i.e., population sizes of neighbouring intervals are shrunk to be equal). In other words, a shrinkage prior automatically penalises for model complexity and practically reduces the model to the amount of variation supported by the data. Thus, shrinkage prior distributions allow the model to be flexible to capture variation in the population sizes through time, while shrinking towards a more constant process in the absence of data. The constant population size and i.i.d. per interval prior distribution can be seen as the two ends of a continuum; the former employs strict shrinkage towards a constant rate process, while the latter employs no shrinkage. In the following subsections, we describe several commonly used prior distributions for Bayesian skyline plots. The mentioned prior distributions are then applied to the StairwayPlot approach.

#### 2.2.1 Independent and Identically Distributed (i.i.d.) Population Sizes

The simplest prior distribution is to assume that each population size $\theta_{i}$ is independent and identically distributed (*i.i.d*.). In principle, there are many possibilities for choosing an *i.i.d*. prior distribution, with the only restriction that the prior distribution must be defined for positive real numbers. Here, we choose three example *i.i.d*. prior distributions. First, the simplest *i.i.d*. prior distribution is the uniform distribution,

(13) $$\theta_{i}∼\text{Uniform}\;\left(0,\;0.1\right)$$

which we define with a minimum of 0 and a maximum of 0.1. The maximum is somewhat arbitrary, but motivated that θ=4Nμ is unlikely to be larger than 0.1.

Second, we defined a lognormal *i.i.d*. prior distribution (UCLN, uncorrelated lognormal) with

(14) $$\begin{array}{l} \mu ∼\text{Loguniform}\;\left(10^{-10},\;100\right)\\ \sigma ∼\text{Exponential}\;\left(1.0/\left(3^{*}H\right)\right)\\ \theta_{i}∼\text{Lognormal}\;\left(\mu ,\;\sigma \right)\; \end{array}$$

Importantly, our lognormal *i.i.d*. prior distribution has estimated mean and standard deviation parameters with corresponding hyperprior distributions. We arbitrarily assigned a loguniform hyperprior distribution for the mean parameter and an exponential hyperprior distribution with mean $3^{*}H$ for the standard deviation parameter, where H=0.587405 because the 95% central probability interval of a lognormal distribution with a standard deviation of SD=0.587405 spans exactly one order of magnitude (Höhna et al. 2017).

Third, we defined a gamma *i.i.d*. prior distribution (IGR, independent gamma rates), inspired from the phylogenetic relaxed clock literature (Drummond et al. 2006), by

(15) $$\begin{array}{l} m∼\text{Loguniform}\;\left(10^{-10},\;100\right)\\ \;v∼\text{Exponential}\;\left(100.0\right)\\ \theta_{i}∼\text{Gamma}\;\left(\frac{m^{2}}{v},\frac{m}{v}\right)\; \end{array}$$

Here, we specified again hyperprior distributions on the mean and variance of the gamma distribution. Specifically, we assigned a loguniform hyperprior distribution for the mean parameter and an exponential hyperprior distribution with mean 0.01 for the variance parameter.

#### 2.2.2 Bayesian Skyline Plot Model

The popularity of the *Bayesian Skyline Plot* model (Drummond et al. 2005) over the previous skyline plot models originates in its ability to provide a smoother demographic inference because of grouping population size parameters between neighbouring intervals together. Each group consists of several consecutive population size parameters which get assigned the same population size value. The number of groups is defined a priori but the number of intervals within each group and the population size for each group is inferred. The number of population size intervals per group is drawn from a flat multinomial distribution with an offset of one (i.e., at least one interval per group). In our analyses, we arbitrarily specified the number of groups as $b=\text{round}\;\left(\frac{2m}{4}\right)$ where m is the number of diploid individuals, i.e., the number of intervals, thus assuming a priori on average four intervals per group.

The Bayesian Skyline Plot assumes autocorrelated, exponentially distributed population size groups,
(16)

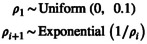




The exponential distribution to model autocorrelation might appear as an unconventional choice, and other models, such as normal and lognormal distributions, to model autocorrelation are possible (Billenstein and Höhna 2024).

#### 2.2.3 Reversible‐Jump Markov Chain Monte Carlo (RJ‐MCMC) Approach

The Bayesian Skyline Plot model introduced the appealing feature to group population size parameters together; however, the Bayesian Skyline Plot model requires an a priori specified number of groups. An alternative approach is to use RJ‐MCMC (Green 1995) to infer the number of groups (similar to the Bayesian stochastic variable search algorithm, Heled and Drummond 2008). Here, we used the RJ‐MCMC algorithm as previously implemented in RevBayes by Freyman and Höhna (2018). We assume that the first population size parameter is drawn from a uniform distribution. The consecutive population size parameters are either equivalent to the value of the preceding group with probability β=0.5, or drawn from an independent uniform distribution. Thus, the number of groups follows a binomial distribution with parameter β. Note that the parameter β can be specified to other values, for example, $\beta =\exp\left(\frac{\ln\left(0.5\right)}{n}\right)$ if one assumes a priori one group with probability 0.5. The RJ prior model is given by
(17)

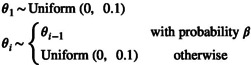




#### 2.2.4 GMRF Model

Autocorrelation between consecutive population size parameters $\theta_{i}$ can be modelled using a GMRF model (Gill et al. 2013; Magee et al. 2020). The GMRF model defines normally distributed variables centered on the previous value. Here, we assume that the log‐transformed populations size parameters $\theta_{i}$ are normally distributed centered on the previous populations size parameter,
(18)

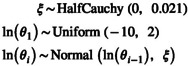

where the global standard deviation parameter ξ governs the amount of variation. ξ can either be estimated (see Gill et al. 2013) or fixed to assume a given total variation, e.g., one order of magnitude, from one side to the other side of the timeline (Magee et al. 2020).

The GMRF model can also be applied using a second order Markov Random Field (GMRF2, Faulkner et al. 2020) where the log‐transformed population sizes are autocorrelated and normally distributed but depend on the previous two instead of one intervals,
(19)

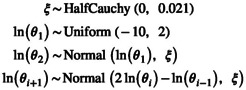




#### 2.2.5 HSMRF Model

The GMRF model assumes the same variation for each time interval a priori. This assumption does not match well in scenarios with few rapid changes but otherwise constant periods, such as population bottlenecks. The HSMRF model (Faulkner et al. 2020; Magee et al. 2020) is an extension to the GMRF model where each interval has its own standard deviation parameter $\xi_{i}$,
(20)

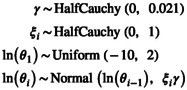




Note that this version of HSMRF uses a global variation parameter γ together with each per interval standard deviation $\xi_{i}∼\text{HalfCauchy}\;\left(0,\;1\right)$ for easier MCMC exploration and is equivalent to using each per interval standard deviation $\xi_{i}∼\text{HalfCauchy}\;\left(0,\;0.021\right)$ instead.

As with the GMRF model, the HSMRF model can also be applied using a second order Markov Random Field (HSMRF2) where the log‐transformed population sizes are autocorrelated and normally distributed but depend on the previous two instead of one intervals,
(21)

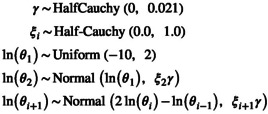




### 2.3 Plotting Demographic Histories

Our goal for StairwayPlot analyses in RevBayes is to display the demographic history over time. In order to visualise the inferred posterior distribution of the demographic history, we need the effective population size and interval times. Recall that the StairwayPlot model implemented in RevBayes is parameterized with the parameters $\theta_{k}=4N_{k}\mu$. Furthermore, the model does not take the interval times as input parameters but assumes them indirectly (Equation 2). We can directly compute the interval times from the parameters θ, as shown by Liu and Fu (2015) by

(22) $$T_{i}=\sum_{k=i}^{n}\frac{\theta_{k}}{k\left(k-1\right)}$$

Similarly, given a mutation rate μ, which we assume to be in units of generations per time, we can compute the per interval effective population sizes

(23) $$N_{k}=\frac{\theta_{k}}{4\mu }$$

These transformations are performed directly in RevBayes and the MCMC samples of the transformed parameters stored for further post‐processing and plotting.

We implemented simple post‐processing and plotting routines into the R‐package RevGadgets (Tribble et al. 2022). RevGadgets first summarises the MCMC samples by (1) defining a fine grid of intervals for plotting, e.g., 500 grid points, (2) breaking each MCMC sampled demographic history (stepfunction obtained from the time intervals and effective population size) into the grid, and (3) computing the 95% credible interval and posterior median (or mean). RevGadgets then constructs a ggplot object that can be used for easy manipulation and plotting (Wickham 2016).

### 2.4 Model Selection

Model selection in Bayesian inference is commonly performed via Bayes factors (Kass and Raftery 1995). Bayes factors compute the ratio of marginal likelihoods, i.e., the probability of the data integrated over all parameter values weighted by their prior distributions, between two competing hypothesis. An emerging alternative approach for model selection in Bayesian inference is cross‐validation, recently advocated for by Lartillot (2023) in phylogenetics. Here, we choose and implement cross‐validation because of its robustness to the underlying prior distribution compared with marginal likelihoods, which can be very sensitive to specific choices of (hyper‐) prior distributions. We are not interested in which prior distribution described the process of population size changes best; instead, we are interested which prior choice enabled us to fit the model best to describe the observed data.

Cross‐validation assesses the fit of a model by using a subset of the data to estimate parameters (i.e., train the model) and the remaining observations to evaluate the predictive fitness. In the most extreme case of leave‐one‐out cross‐validation, we use all but one observation to estimate the parameters, $P\left(\theta |X_{\left(-i\right)}\right)$, then compute the probability of observing the removed observed, $P\left(X_{i}|\theta \right)$, integrate over all parameters, $P\left(X_{i}|X_{\left(-i\right)}\right)=\int p\left(X_{i}|\theta \right)p\left(\theta |X_{\left(-i\right)}\right)\mathrm{d\theta}$, and repeat this process for all data points.

Fortunately, Lewis et al. (2014) and Lartillot (2023) developed an approach were MCMC samples of the whole dataset can be used to perform leave‐one‐out cross‐validation instead of running one MCMC simulation per data subset. Specifically, we compute the log‐transformed posterior cross‐validation (LPCV) score as

(24) $$\begin{array}{c} \text{LPCV}=\sum_{i=1}^{N}\ln\left(P\left(X_{i}|X_{\left(-i\right)}\right)\right)\\ \;=\sum_{i=1}^{N}\ln\left(\int p\left(X_{i}|\theta \right)p(\theta |X_{\left(-i\right)})\mathrm{d\theta}\right)\\ \;=\sum_{i=1}^{N}\ln{\left(\int \frac{1}{p\left(X_{i}|\theta \right)}p\left(\theta |X\right)\mathrm{d\theta}\right)}^{-1}\\ ≃\sum_{i=1}^{N}\ln{\left(\frac{1}{K}\sum_{k=1}^{K}\frac{1}{p\left(X_{i}|\theta_{k}\right)}\right)}^{-1}\; \end{array}$$

In the first step, we defined the LPCV as the sum of the individual log‐transformed leave‐one‐out cross‐validation scores $P\left(X_{i}|X_{\left(-i\right)}\right)$. In the second step, we expand the individual leave‐one‐out cross‐validation scores by the integral over the parameter values, $P\left(X_{i}|X_{\left(-i\right)}\right)=\int p\left(X_{i}|\theta \right)p\left(\theta |X_{\left(-i\right)}\right)\mathrm{d\theta}$. The third step replaces the posterior distribution given a subset of the data with the posterior distribution of the full data. The final step replaces the integral over the posterior distribution by the sum over the MCMC samples. More details about the original derivation can be found in Lewis et al. (2014) and Lartillot (2023).

In the StairwayPlot model, $X_{i}$ refers to the frequency of a single site and $p\left(X_{i}|\theta_{k}\right)$ to the site frequency probabilities (Equation 4). We only need to store per MCMC sample the site frequency probabilities to compute the LPCV. The posthoc computation of the LPCV after the MCMC analysis is done extremely efficient.

### 2.5 Implementation

We implemented our Bayesian StairwayPlot model in the software RevBayes (Höhna et al. 2016). RevBayes is an open‐source software https://github.com/revbayes/revbayes written in C++. Documentation about RevBayes is available from https://revbayes.github.io. A specific tutorial about StairwayPlot analysis using RevBayes is found at https://revbayes.github.io/tutorials/stairwayplot.

### 2.6 Simulation Study

We evaluated the implementation of our Bayesian StairwayPlot model in RevBayes using a set of standard single population simulations originally created and obtained from Liu and Fu (2015). These simulation scenarios included (a) a constant‐size model, (b) a two‐epoch model, (c) an exponential growth model without earlier constant period, (d) an exponential growth model with an earlier constant period, (e) another expansion model (complex model) and (f) a bottleneck scenario (standard model). The original simulations were performed using the software ms (Hudson 2002) using a default mutation rate (μ) of 1.2 × 10^−8^ per bp per generation and a recombination of ρ=0.8θ per bp per generation. Note that 200 simulations were performed and the final SFS was computed as the average of the replicates. For each of these datasets, we performed an MCMC simulation with 500,000 iterations and four replicated runs (to check for convergence) for each of the nine prior models (see Equations ((13), (14), (15), (16), (17), (18), (19), (20), (21)–(13), (14), (15), (16), (17), (18), (19), (20), (21)) above). Additionally, we used StairwayPlot2 (Liu 2020) on the same datasets as a reference. We plotted the resulting demographic histories against the ‘true’ demographic history using RevGadgets (Tribble et al. 2022).

We extended the simulation study to explore the impact of wrongly assuming derived/ancestral alleles. Specifically, for each SNP, we placed it with probability $\rho \in \left\{0.0,\;0.001,\;0.005,\;0.01,\;0.05,\;0.1\right\}$ into the opposite bin, i.e., the bin n−i instead of i. We then inferred the demographic history for these six different true demographic histories and six different error probabilities. However, for simplicity, we only used one prior model for these inferences.

### 2.7 Empirical Case Studies

As an empirical exploration, we used the recently published firefly dataset from Catalan et al. (2024). For our exploration, we chose the Munich population of the species *Lampyris noctiluca* (big European firefly). The dataset consists of 20 diploid individuals from which whole genome resequencing data was obtained (full genome size approx. 633 MB with 6,557,599 SNPs).

We hope that this dataset serves as a good case study to elaborate specific modelling and inference choices. In our first analysis, we applied all nine prior models for our Bayesian StairwayPlot analysis and additionally used StairwayPlot2 (Liu 2020) for a comparison. Then, we chose the best prior model and performed six additional explorations testing the impact of different modelling and data acquisition choices. (i) The first additional exploration targets the derivation of the site frequency probability of monomorphic sites (Equations (5) and (7)). This is a purely methodological exploration and is irrespective of the dataset. (ii) The second exploration focuses on the sequence lengths, from which the SNPs were obtained. Commonly, the SNPs are obtained based on a reference genome, but later on filtered for different reasons (e.g., removing low‐coverage SNPs and triallelic sites). This filtering step makes it difficult to assess how many monomorphic sites are truly represented by the sequence. (iii) The third exploration focuses on omitting monomorphic and/or singleton sites. Specifically, we explored whether removing monomorphic sites (Equation (9)) or merging singleton sites with monomorphic sites (Equation (10)) impacts the inferred demographic history. (iv) The fourth exploration targets the impact of the assumed mutation rate, specifically if uncertainty in the mutation rate can be integrated using a distribution. (v) The fifth exploration focuses on the power of our Bayesian StairwayPlot approach when fewer individuals are sequenced, e.g., only 10 or 15 instead of 20. (vi) The sixth exploration focuses on the power of our Bayesian StairwayPlot approach when fewer sites are available, e.g., when not whole genome sequencing was performed.

## 3 Results

### 3.1 Simulation Study

The likelihood function of the StairwayPlot was approach originally derived by Liu and Fu (2015) and implemented in a maximum likelihood framework (Liu and Fu 2015, 2020; Liu 2020). Here, we implemented the exact same likelihood function in a Bayesian inference framework in the software RevBayes (Höhna et al. 2016). Our implementation enables us to use different prior models to infer population size change through time akin to the popular Bayesian skyline plots.

**FIGURE 1:**
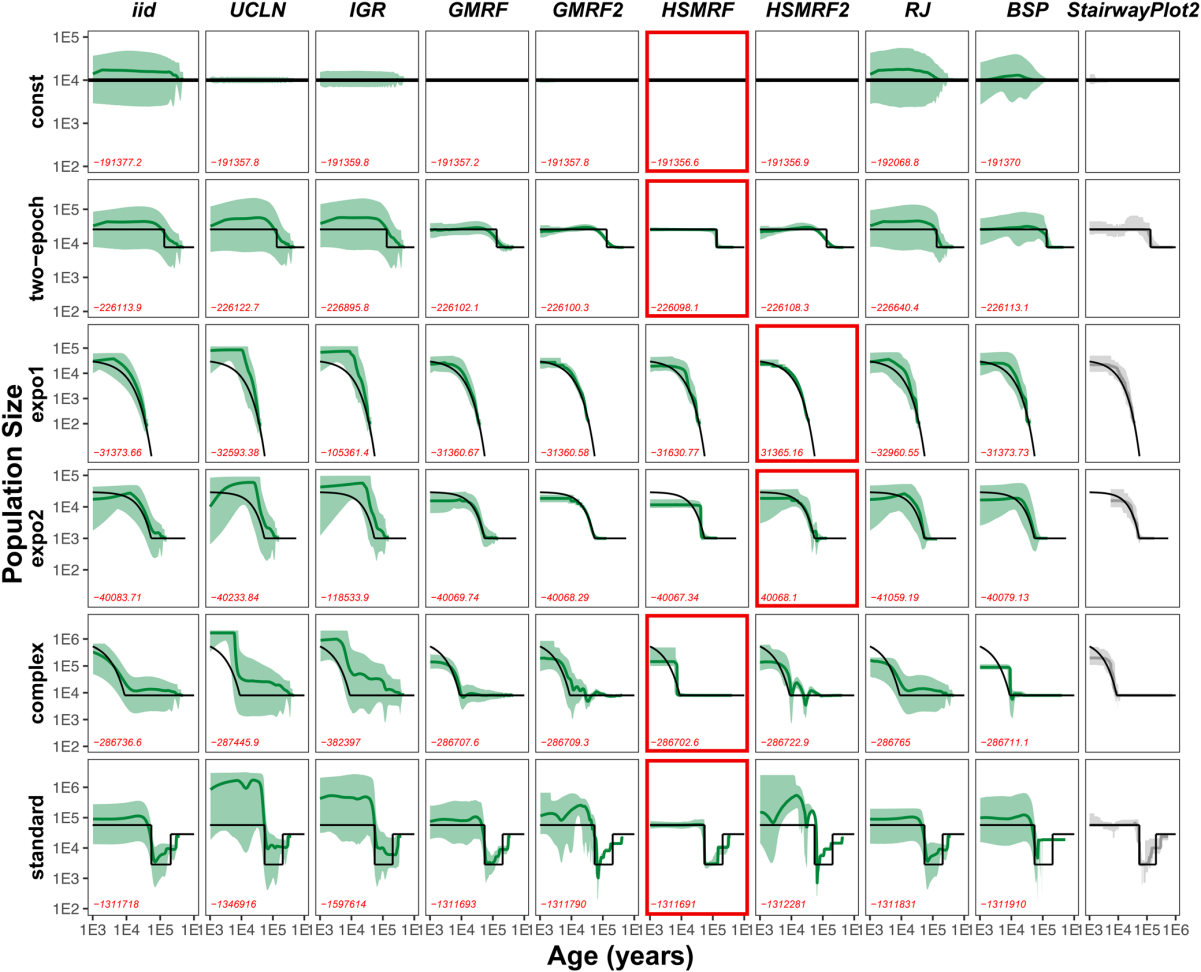
Demographic histories estimated using our Bayesian StairwayPlot approach with different prior models for several standard simulation scenarios. Each plot shows the 95% credible interval (shaded light green area), the posterior median demographic history (solid dark green line), the true demographic history (solid black line) and the leave‐one‐out cross‐validation probability (red numbers). We used the nine prior models as described in the main text and the original StairwayPlot2 implementation (Liu 2020) as a reference (light grey). The best model according to the leave‐one‐out cross‐validation probability is highlighted in red.

Our exploration using the simulated data from standard models (originally produced by Liu and Fu 2015) showed very good performance comparable to the original implementation (Figure 1). Our best performing model showed matching performance to the original StairwayPlot2 implementation in the median estimate while showing a narrower, i.e., more precise, 95% credible interval. The selection of the best model using the leave‐one‐out cross‐validation probability matches our visual selection of the model that comes closest to the true underlying simulation scenario (Figure 1).

From our nine prior models, the first and second order HSMRF and HSMRF2 performed best according to the leave‐one‐out cross‐validation probability. The first order HSMRF performed better when the true simulation scenario included abrupt changes in population sizes. This result is expected as the HSMRF is designed for scenarios with few large jumps between longer periods of stasis. The second order HSMRF performed better when the true simulation scenario included continuous changes in population size (the two exponential population growth simulation). Again, this behaviour is expected as the second order HSMRF produces more smooth and continuous trajectories (Faulkner et al. 2020). Overall, different models will perform better depending on the ‘true’ demographic history, and our efficient leave‐one‐out cross‐validation probability can select the best fitting model.

Our exploration of the impact of wrongly assuming derived/ancestral alleles showed that, as expected, the inferred demographic history becomes more biased with higher error probabilities (Figure S8). Small amounts of errors in assuming derived/ancestral alleles (i.e., less than 0.01 probability of an error per SNP) did not impact the inferred demographic history, while larger errors, i.e., with an error probability of 0.01 per SNP or higher, lead to artificial variation in the inferred demographic history. Thus, if ancestral/derived alleles cannot be assigned with 99% certainty or higher, it is advisable to use folded site frequency spectra instead.

### 3.2 Empirical Case Study: The Demographic History of the Big European Firefly

The demographic history of the big European firefly ( *Lampyris noctiluca* ) is unknown and likely more complex than the simulation scenarios explored above, with more extreme and rapid variation in population size, especially due to the impact of the last ice age (Catalan et al. 2024). We expect that the big European firefly, which today is found in almost all European countries, had a smaller distribution and range during the last ice age (approx. 115,000–11,700 years ago) and therefore also a smaller population size during this time. After the end of the last ice age, we expect that the big European firefly has expanded in range and therefore also increased in population size. However, due to recent increase in human population size and its impact on natural habitats, we speculate that the big European firefly has decreased in population size recently.

**FIGURE 2:**
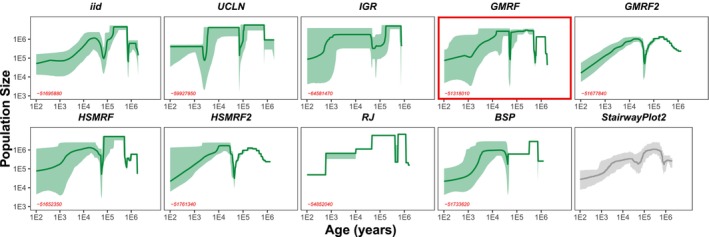
Demographic histories estimated using our Bayesian StairwayPlot approach with different prior models for the big European firefly. Each plot shows the 95% credible interval (shaded light green area), the posterior median demographic history (solid dark green line) and the leave‐one‐out cross‐validation probability (red numbers). We analysed the *Lampyris noctiluca* population data from Munich (Catalan et al. 2024). We used the nine prior models as described in the main text and the original StairwayPlot2 implementation (Liu 2020) as a reference (light grey). The best model according to the leave‐one‐out cross‐validation probability is highlighted in red.

Our Bayesian StairwayPlot analyses inferred a highly variable demographic history of the big European firefly (Figure 2). The nine different prior models plus the maximum likelihood analysis using StairwayPlot2 showed alternating periods of higher and lower population size, overall declining towards the present. The effective population size towards the present seems to have decreased towards 80,000–100,000, while the maximum effective population size during the entire history was ∼7,300,000.

Most models inferred a brief population bottleneck about 90,000 years ago and possibly a second more ancient bottleneck almost 1 million years ago. The first bottleneck falls into the last ice age, although the rebound occurred before the end of the last ice age. If we were to trust our time estimates, then this rebound implies that the big European firefly has quickly stabilised and recovered, e.g., due to adaption to the new harsh environment of the last ice age. However, our time estimates fundamentally depend on the assumed mutation rate. If the true mutation rate was higher than our estimate, then the true time of the bottleneck would have been more recent. Interestingly, in our exploration, where we applied prior uncertainty on the mutation rate, we observed that the inferred demographic history becomes more smooth (Figure S1). Thus, not knowing the mutation rate precisely erodes the magnitude of population size changes and smears the population size changes over time, effectively rendering the population size change undetectable (Figure S1). It might be more advisable in the case of mutation rate uncertainty to perform several analyses with fixed but different mutation rates.

Another factor that influences both the time of population size changes and the absolute population size is the number of monomorphic sites. In many empirical situations, the total sequence length is at best a good guess. The total sequence length should include all SNPs and all monomorphic sites. However, since often sites are filtered out, e.g., because they are triallelic instead or biallelic or because of repetitive elements, the true total sequence length is challenging to compute. In our exploration of the impact of the total sequence length, we found that the observed pattern of the demographic history is unaffected (Figure S2). The timing of population size changes, e.g., inferred bottlenecks, is shifted based on the assumed number of monomorphic sites. A 10‐fold larger number total sequence length shifts these event 10‐times more recent, and similarly down‐scales the population size by a factor of 10 (Figure S2). This result might not be surprising from the underlying coalescent theory; nevertheless, we found it important to highlight this impact for empirical studies.

Not knowing the number of monomorphic sites and thus conditioning on only having observed SNPs complicated our analyses, as multiple models failed to produce sensible results. When we obtained sensible results, the inferred demographic history showed the same pattern as when including monomorphic sites (Figure S3). The main distinction was a slight shift towards higher absolute population size estimates and more ancient population size changes, in line with our exploration when assuming a shorter total sequence length (Figure S2). We found that excluding singletons (i.e., including SNPs that where found only in one individual into the monomorphic site category) had no discernible impact on the inferred demographic history, except that the most recent population size change cannot be detected (Figure S3). This result is expected as singletons represent mutations on terminal lineages, thus affecting the most recent coalescent event. Not having this data naturally complicates estimates in the most recent time. Furthermore, we observed that computing the probability of a site falling into the monomorphic category, either using the total expected tree length or simply one minus the probability of all categories, produced virtually the same inferred demographic history (Figure S4). Overall, the two different approaches of computing the probability of observing a monomorphic site are very similar and only start deviation more for larger values of θ (Figure S5).

Our final exploration focused on the number of individuals and fraction of the whole genome used for demographic inference. We found that using the whole genome (approx. 633 MB yielding 6,557,599 SNPs) or 10% of the genome showed the best performance in terms of recovering the full complex demographic history (Figure S5). Using 1% of the genome or less was not able to recover the full variation in population size over time. On the other hand, the number of individuals showed a smaller impact on the inferred demographic history (Figure S6). As few as 10 diploid individuals retained sufficient information to capture the variable demographic history, while using only five individuals appeared to be not sufficient (Figure S6).

## 4 Discussion and Conclusions

The StairwayPlot approach provides a powerful likelihood‐based inference for complex demographic histories of varying population sizes. Here we implemented the same likelihood function as in the original StairwayPlot2 software (Liu and Fu 2015, 2020; Liu 2020), which uses maximum likelihood, into a Bayesian inference framework in the software RevBayes (Höhna et al. 2016).

One major strength of the StairwayPlot approach is that the likelihood is extremely fast to compute; it takes only a few seconds to compute the likelihood 1 million times for the big European firefly example. The computational time does not depend on the number of SNPs but instead on the number of sequenced individuals (we used 20 diploid individuals in our example). Thus, the StairwayPlot approach can be applied easily to whole genome data and the fast likelihood computation makes model exploration even on a standard desktop or laptop computer feasible.

Our implementation in RevBayes enables the full flexibility of specifying, exploring and comparing different prior models. The key advantage of our RevBayes implementation is the probabilistic graphical model foundation (Höhna et al. 2014) where the StairwayPlot likelihood is used as an observed stochastic variable (the data). The parameters on which the StairwayPlot likelihood depends can either be fixed, or any prior distribution can be assigned. Specifically, we demonstrated and explored here common prior distributions used for demographic inference in Bayesian skyline plot approaches (Drummond et al. 2005; Ho and Shapiro 2011; Billenstein and Höhna 2024).

In our exploration, we found that the more complex prior models, namely, the first and second order HSMRF and HSMRF2, performed best on the simulated data. For the empirical big European firefly dataset (Catalan et al. 2024), we found that GMRF performed best. For future empirical studies, it might not be necessary to explore the full range of different prior models, although this is clearly computationally feasible. For example, it might be sensible to omit the independent prior models UCLN and IGR as well as the reversible‐jump MCMC and grouped intervals Bayesian skyline plot model, because these performed worst in the simulations.

All of our analyses used the exact same underlying likelihood, the StairwayPlot likelihood. Although the only difference are the prior distributions, the inferred demographic histories varied considerably (Figure 2). Fortunately, model selection using the leave‐one‐out cross‐validation probability was able to identify the best matching demographic history in the simulations. Most importantly, our implementation of the leave‐one‐out cross‐validation probability can be computed extremely efficiently from the MCMC samples. Therefore, we recommend to explore at least several different prior models and to choose the most supported model based on the leave‐one‐out cross‐validation probability.

Our results about the sensitivity of the assumed prior model agree with the often observed uncertainty or difficulty in estimating demographic histories from site frequency spectra alone (e.g., Deng et al. 2024). The information used by site frequency based approaches is limited and multiple demographic histories can yield closely matching expected site frequency spectra (Myers et al. 2008; Bhaskar and Song 2014; Terhorst and Song 2015). Therefore, we recommend, if possible, to explore additionally alternative approaches that use linkage information in the genetic sequences, such as approaches based on the sequential Markovian coalescent (Li and Durbin 2011; Schiffels and Durbin 2014; Terhorst et al. 2017) or ancestral recombination graphs (Rasmussen et al. 2014; Kelleher et al. 2019; Brandt et al. 2022; Nielsen et al. 2024).

Finally, we observed several important aspects for empirical studies: (1) incorporating uncertainty in the mutation rate erases the clear signal in the demographic history, and thus it is advisable to explore several fixed mutation rate estimates; (2) the total sequence length, as the mutation rate, is crucial to obtain unbiased estimates of the absolute population size and the timing of population size changes; (3) we advise to apply the folded site frequency spectrum if ancestral/derived alleles cannot be called at a 0.99 probability or higher; (4) it is more important to invest in good quality whole genome datasets with at least several hundred thousand SNPs and even 10 individuals are sufficient.

## Author Contributions

S.H. and A.C. designed research, S.H. implemented the method and performed the analysis and S.H. and A.C. wrote the paper.

## Conflicts of Interest

The authors declare no conflicts of interest.

## Supporting information


Data S1.

## Data Availability

Data sharing is not applicable to this article, as no new data were created. The data analysed in the simulation study were retrieved from Liu and Fu (2015) and Liu and Fu (2020) (downloaded from https://zenodo.org/records/3958302). The data for the empirical exploration were retrieved from Catalan et al. (2024).
