# Supplementary material for: Bayesian StairwayPlot for Inferring Single Population Demographic Histories From Site Frequency Spectra
Source: Mol Ecol Resour. 2025 Feb 26;25(6):e14087. doi: 10.1111/1755-0998.14087 (PMC12225710; doi:10.1111/1755-0998.14087)
Supplement: Supplementary file 1 — Data S1. [file MEN-25-e14087-s001.pdf]

Bayesian StairwayPlot for Inferring Single Population Demographic Histories from Site Frequency Spectra

Supplementary Material

Sebastian Höhna[1,2] and Ana Catalán[3]

[1] *GeoBio-Center, Ludwig-Maximilians-Universität München, 80333 Munich, Germany*
[2] *Department of Earth and Environmental Sciences, Paleontology & Geobiology, Ludwig-Maximilians-Universität München, 80333 Munich, Germany*
[3] *Division of Evolutionary Biology, Ludwig-Maximilians-Universität München*

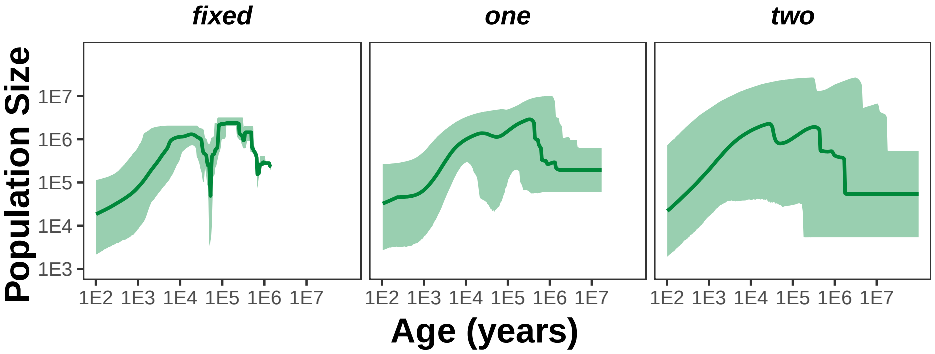

Figure S1: Exploration of the mutation rate assumptions on the inferred demographic history. The demographic history was inferred for the Munich population of the big European firefly (*Lampyris noctiluca*, Catalan *et al.* 2024). We assumed the mutation rate either to be known exactly (left, $\mu = 2.8E - 9$), with a prior of one order of magnitude uncertainty (middle, $\mu \sim \text{lognormal}(\text{mean} = 2.8E - 9, \text{sd} = 0.587405)$), and with a prior of two orders of magnitude uncertainty (middle, $\mu \sim \text{lognormal}(\text{mean} = 2.8E - 9, \text{sd} = 2 * 0.587405)$). We observe strong population size variation only if the mutation rate was assumed to be fixed. The more uncertainty in the mutation rate was assumed, the more the population size changes go smeared over time and became unrecognizable.

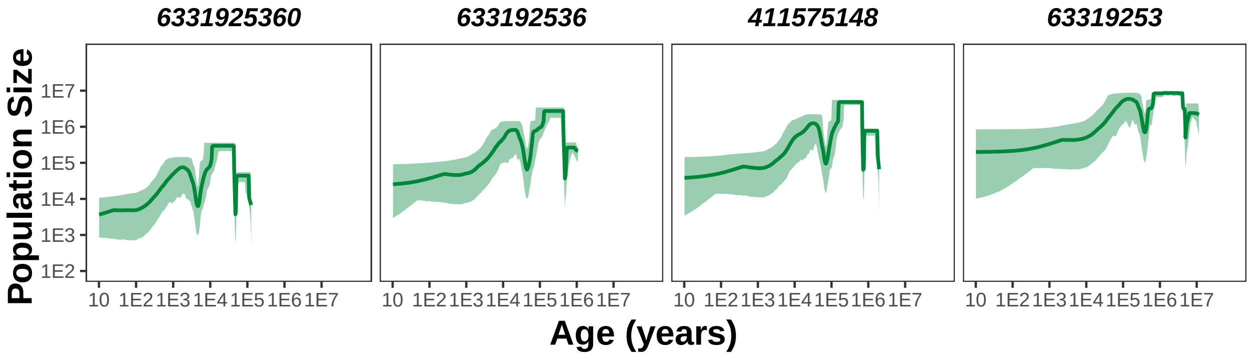

Figure S2: Exploration of the assumed sequence length on the inferred demographic history. The demographic history was inferred for the Munich population of the big European firefly (*Lampyris noctiluca*, Catalan *et al.* 2024). The assumed total sequence length (number of SNPs plus number of monomorphic sites) were from left to right: 10-times the total genome size, the actual genome size (633MB), 65% of the actual genome size to reflect filtering of sites, and 10% of the genome size. The general pattern of variation in population size was the same for all assumed total sequence lengths. The main difference is timing of the population size changes and absolute values of the population size. In general, an x-fold larger total sequence length while keeping the number of SNPs the same resulted in x-fold earlier population size changes and x-fold smaller absolute population size.

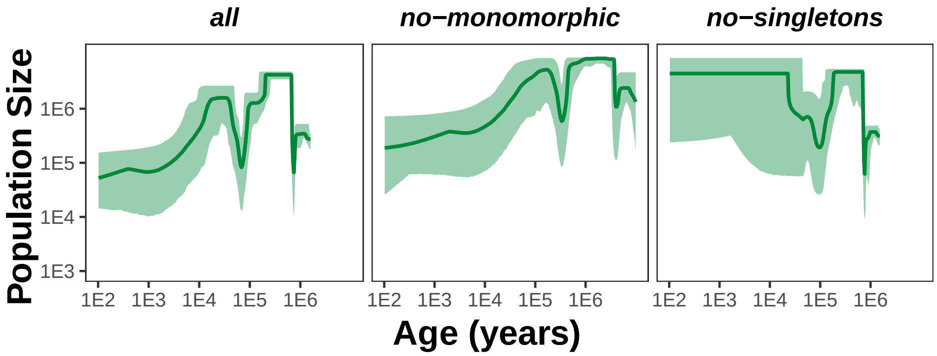

Figure S3: Exploration of the ascertainment bias correction for not using monomorphic or singleton sites. The demographic history was inferred for the Munich population of the big European firefly (*Lampyris noctiluca*, Catalan *et al.* 2024). We either used *all* available sites, excluded monomorphic sites and applied the corresponding ascertainment bias correction (*no-monomorphic*), or combined singletons and monomorphic sites (*no-singletons*). The results show that the general pattern, especially deeper in the past, was the same for all model assumptions. The *no-singletons* condition removes information in the most recent past, as singletons provide information about the coalescent time of 2 of the N individuals. The condition *no-monomorphic* sites shifted the absolute population size upwards and the population size changes back in time (see also Figure S2).

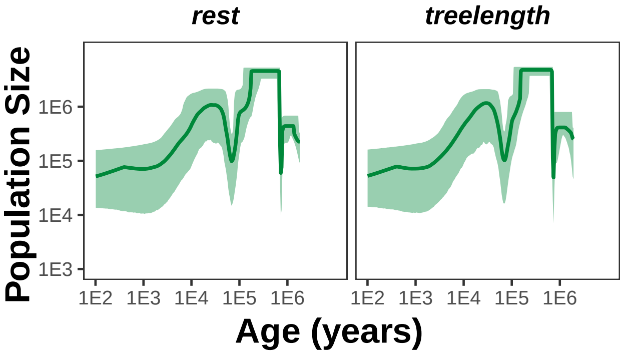

Figure S4: Exploration of the theoretical computation for the probability of monomorphic sites. The demographic history was inferred for the Munich population of the big European firefly (*Lampyris noctiluca*, Catalan *et al.* 2024). On the left side, we show the demographic history inferred when computing $p_0 = 1 - \sum_{i=1}^{n-1} p_i$. In the right side, we show the demographic history inferred when computing $p_0 = e^{-\sum_{k=2}^{n} \frac{\theta_k}{(k-1)}}$. Both approaches lead to the same inferred demographic history, indicating that using either the rest or tree-length to compute the probability of monomorphic sites is viable.

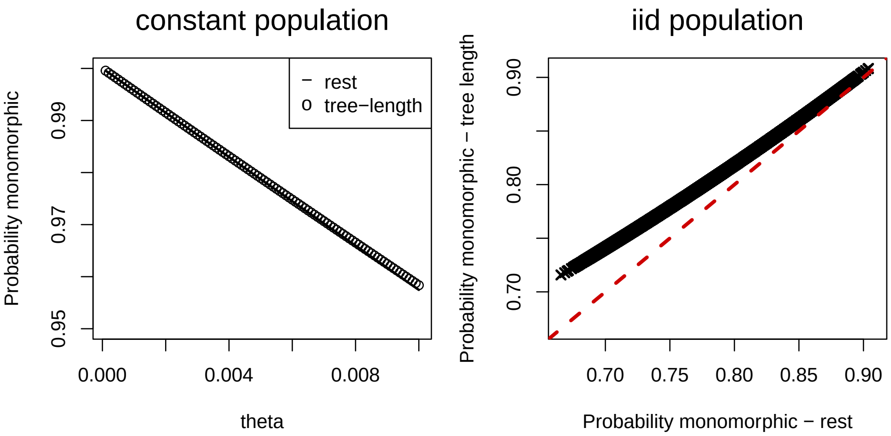

Figure S5: Exploration of the theoretical computation for the probability of monomorphic sites. We focus on the comparison between computing p_0 as the remaining probability (rest, $p_0 = 1 - \sum_{i=1}^{n-1} p_i$) and using the tree length($p_0 = e^{-\sum_{k=2}^{n} \frac{\theta_k}{(k-1)}}$). As data we used the Munich population of the big European firefly (*Lampyris noctiluca*, Catalan *et al.* 2024). On the left side, we show the difference in the compute p_0 values for different θ parameters under a constant population size demography model. On the right side we randomly drew 1000 configurations of iid θ values and contrast directly p_0 under the two approaches. While the p_0 are very similar, they start diverging for larger values of θ, which is also seen by smaller probabilities of p_0.

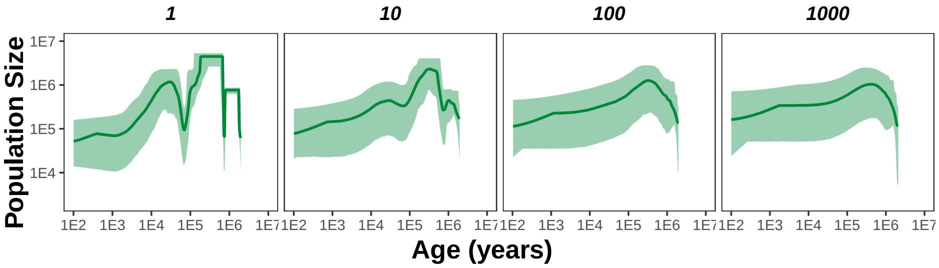

Figure S6: Exploration of the data thinning, i.e., fewer used SNPs, on the inferred demographic history. The demographic history was inferred for the Munich population of the big European firefly (*Lampyris noctiluca*, Catalan *et al.* 2024). From left to right, we only sampled: every site in the genome, every 10th site, every 100th site and every 1000th site. The total sequence length was adjusted accordingly in the analyses. Our full dataset contained 6,557,599 SNPs. Thus, using all SNPs gives strikingly the best demographic history, while already 1% of SNPs, e.g., 65,576 SNPs was not sufficient to recover the complex demographic history.

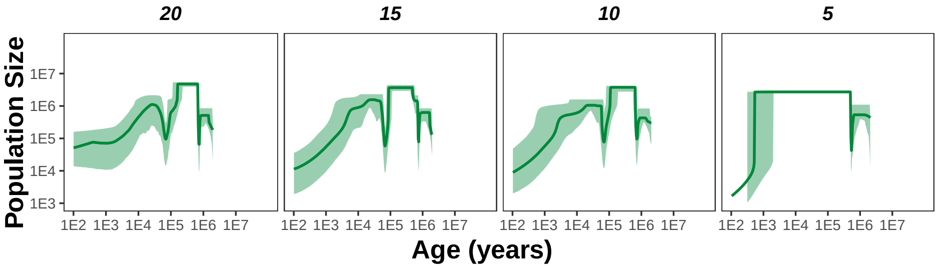

Figure S7: Exploration of the data thinning, i.e., fewer used individuals, on the inferred demographic history. The demographic history was inferred for the Munich population of the big European firefly (*Lampyris noctiluca*, Catalan *et al.* 2024). From left to right, we only sampled: all 20 diploid individuals, 15 individuals, 10 individuals or 5 individuals. The overall pattern in population size changes was the same for 10 or more sample individuals. Using only 5 individuals seem not to be sufficient to recover the complex demographic history.

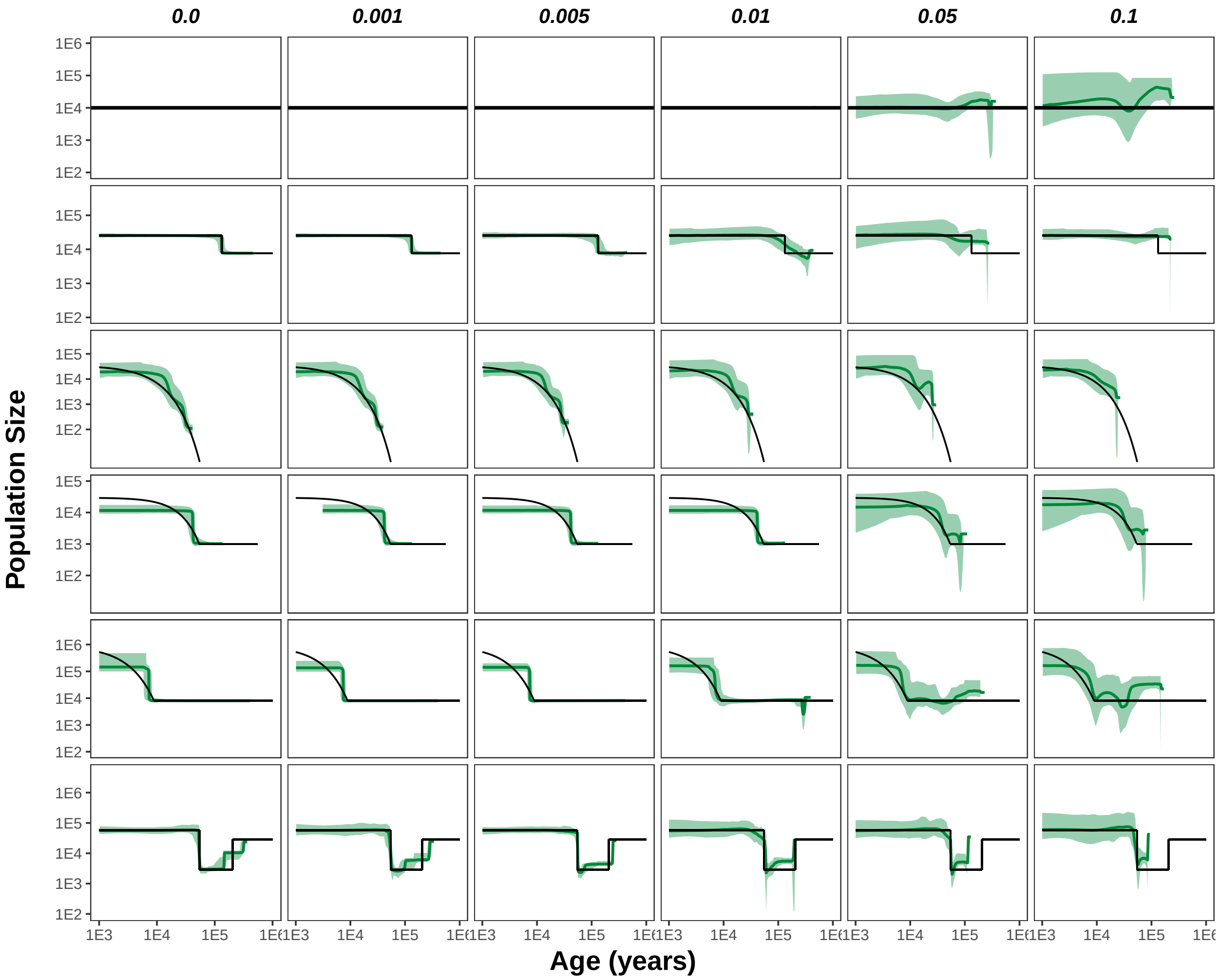

Figure S8: Exploration of the impact of wrongly calling ancestral vs. derived alleles on the inferred demographic history. Each plot shows the 95% credible interval (shaded light green area), the posterior median demographic history (solid dark green line), and the true demographic history (solid black line). We only used the HSMRF model for this exploration. The different rows show different scenarios under which the data were simulated. The different columns show the probability of a side being wrongly coded, i.e., in the second column we placed a site with probability $p = 0.001$ into the opposite bin (switching derived and ancestral for this site). Starting with an error of 0.01 we see biases in our inference, and for errors of 0.1 we obtain spurious results.

References

Catalan, A., Gygax, D., Candolin, U., Tusso, S., Duchen, P. & Höhna, S. (2024) Sex-biased migration and demographic history of the big european firefly lampyris noctiluca. *bioRxiv*, pp. 2024–01.
